# Literary Notes

**Published:** 1906-06

**Authors:** 


### LITERARY NOTES.

Messrs. William Wood & Company, publishers, New York, announce the American Practice of Surgery, edited by Professor Joseph Decatur Bryant, M.D., president of the Medical Society of the State of New York, orator in surgery, American Medical Association 1906, and Dr. Albert Henry Buck, editor of the "Reference Handbook of the Medical Sciences," and other works. This treatise will be published in eight royal octavo volumes, profusely illustrated in colors, line and half-tone engravings. There are ninety-six contributors, and one hundred different titles, in eighteen parts, and a general index to the whole, besides one to each separate volume. The work will be sold by subscription for the full set, each volume costing from seven to nine dollars, according to binding. The first volume will be published in June, and the others will follow at intervals of about three months.

This is the most comprehensive surgical treatise ever projected in this country, and the most distinguished writers have been secured as contributors. Orders should be placed promptly, as it is quite likely the first edition will be exhausted soon after leaving the press.

